# Need for ensuring cultural competence in medical programmes of European universities

**DOI:** 10.1186/s12909-018-1449-y

**Published:** 2019-01-15

**Authors:** Janne Sorensen, Marie Norredam, Jeanine Suurmond, Olivia Carter-Pokras, Manuel Garcia-Ramirez, Allan Krasnik

**Affiliations:** 1 Danish Research Centre for Migration, Ethnicity and Health, Department of Public Health, University of Copenhagen, Oester Farimagsgade 5A, 1014 Copenhagen, Denmark; 2 Academic Medical Center, University of Amsterdam, Department of Public Health, Amsterdam Public Health research institute, P.O. Box 22660, 1100 DE Amsterdam, The Netherlands; 3 School of Public Health, University of Maryland, 4200 Valley Drive, Suite 2242G, College Park, MD 20742-2611 USA; 4 Faculty of Psychology, University of Seville, C/ Camilo José Cela, S/N, 41018 Sevilla, Spain

**Keywords:** Cultural competence, Medical education, Medical teachers, Medical curricula, Ethnic minorities, Immigrants, C2ME, Diversity sensitivity

## Abstract

**Background:**

Europe is becoming more social and cultural diverse as a result of the increasing migration, but the medical doctors are largely unprepared. The medical education programmes and teachers have not evolved in line with development of the population. Culturally competent curricula and teachers are needed, to ensure cultural competence among medical doctors and to tackle inequalities in health between different ethnic groups. The objective of this EU financed study is therefore to provide a snapshot of the role of cultural competence in European medical educational programmes.

**Methods:**

A questionnaire was developed in order to uncover strengths and weaknesses regarding cultural competence in the European medical education programmes. The questionnaire consisted of 32 questions. All questions had an evidence box to support the informants’ understanding of the questions. The questionnaire was sent by email to the 12 European project partners. 12 completed questionnaires were returned.

**Results:**

Though over half of the participating medical programmes have incorporated how to handle social determinants of health in the curriculum most are lacking focus on how medical professionals’ own norms and implicit attitudes may affect health care provision as well as abilities to work effectively with an interpreter. Almost none of the participating medical programmes evaluate the students on cultural competence learning outcomes. Most medical schools participating in the survey do not offer cultural competence training for teachers, and resources spent on initiatives related to cultural competences are few. Most of the participating medical programmes acknowledge that the training given to the medical students is not adequate for future jobs in the health care service in their respective country regarding cultural competence.

**Conclusions:**

Our results indicate that there are major deficiencies in the commitment and practice within the participating educational programs and there are clear potentials for major improvements regarding cultural competence in programmes. Key challenges include making lasting changes to the curriculum and motivating and engaging stakeholders (teachers, management etc.) within the organisation to promote and allocate resources to cultural competence training for teachers.

## Background

As a result of rising immigration, the European Union (EU) is increasingly becoming a culturally diverse place. In 2015, there were 52.8 million (10.4% of the total EU-28 population) people born outside their country of residence [1]. Of these, 34.3 million were born outside the EU. Currently, migration to Europe is dominated by complex migration flows. These are composed of people fleeing from persecution or violence, or seeking a better life away from their homeland. They have often travelled in extreme conditions and are frequently victims of smuggling and trafficking [2]. In addition, migrants and ethnic minority groups living in European countries are constituted by a number of diverse subgroups, including labor-migrants, students, migrants who seek to reunify with their families and undocumented migrants.

Several European studies have documented worse health outcomes for some migrants and ethnic minorities compared to EU native-born residents for a number of diseases, including mental health disorders, non-communicable diseases like diabetes and ischemic heart diseases as well as communicable diseases such as tuberculosis and HIV/AIDS [3–6]. Many migrants and ethnic minorities also have lower self-rated health than the majority population [7]. However, disease patterns vary with different migrant and ethnic minority groups because various factors such as gender and genes are determined by risk factors associated with cultural traditions as well as migrant processes that are frequently associated with the extreme travelling, receiving and settlement conditions [8]. Simultaneously, access to health care services for migrants and ethnic minorities is often impeded by a number of barriers. These barriers are associated with ‘newness’ to the health care system and not knowing how to navigate through it, as well as with language and communication barriers that may lead to delay in help seeking, diagnosis and treatment [9]. Also, low health literacy (the ability to access, understand, appraise and apply health-related information [10]) makes it difficult to navigate through the health care system and may contribute to impeded access to health care for migrants and ethnic minorities [11, 12]. Moreover, some groups of migrants experience legal and other barriers to access quality health care. These barriers are not formal barriers; as such, they must be observed as expressions of systemic institutional discrimination. They entail structural barriers that shape health inequities, which consolidate imbalanced power relations and discrimination against minority groups within the healthcare system and its services [13, 14]. Consequently, one of the great challenges of European immigration is managing migrants and ethnic minorities’ health needs and handling diversity in healthcare systems. If these issues are not addressed, immigration may result in increasing inequalities in health and large health gaps in European countries.

Ensuring cultural competence (CC) among medical doctors is one important strategy to tackle inequalities in health between different ethnic diverse groups [15]. CC is often defined as a set of coherent skills, knowledge and attitudes related to a) knowledge of epidemiology and differential effects of treatment in various ethnic groups, b) skills for dealing with cultural diversity, hereunder communication and c) attitudes such as humility, empathy, curiosity, respect, sensitivity and awareness [16, 17]. Many medical doctors are largely unprepared to meet the special needs of migrants and ethnic minority patients and the delivery of healthcare to this diverse patient population. In a 2014 Dutch study, Seeleman et al. demonstrated that medical doctors and students lack knowledge of CC and culturally competent behaviour, including experience using interpretation services [18].

Even though several initiatives exist to improve the teaching of cultural competence to medical students, studies have shown that while medical schools are generally free to determine their own policies, they fail to address institutional policies focused on mobilising academic communities to embed the study of health care for migrant health and ethnic minorities into the curriculum [19, 20]. A 2010 review of medical schools in the United States (US), United Kingdom (UK) and Canada has shown that all three countries lack conceptual clarity, and have fragmented and variable programmes that do not include support to faculty and staff [21] on these issues.

Existing evidence show gaps and redundancies in training of medical doctors, however do not identify the institutional level where these gaps and redundancies can best be addressed within medical schools. To gain deeper insight into how CC is prioritised this study investigates the level of CC in 12 European university medical education programmes on different institutional levels. This study aims to provide a snapshot of the CC situation in medical educations in Europe in order to call attention to how improvements can be implemented at different institutional levels.

## Methods

### Participants and setting

This study is part of the EU-financed project ‘Culturally Competent in Medical Education (C2ME)’ carried out by 13 partners (12 EU and 1 US) between 2013 and 2015. The partners in the project were chosen on the basis of their expertise in the fields of migrant health and medical education. EU countries have 312 such medical programmes. Our project represents 4% of these, or a total of approximately 20,000 enrolled medical students. The aim of the C2ME project was to support the development of educational training systems that focus on CCs that improve the quality of education and promote equity. The project specifically addressed teaching staff in medical schools and curriculum. Teaching staff in the project is defined as faculty staff with teaching obligations.

### Development of questionnaire and data collection

A questionnaire designed to uncover the strengths and gaps in relation to CC in medical programmes was developed. The questionnaire was inspired by existing questionnaires such as the Tool for Assessing Cultural Competence Training (TACCT) [22] and the self-assessment tool ‘Standards for equity for healthcare for migrant and other vulnerable groups’ [23]. It was designed to target different policy levels within medical schools, including organizational and strategic elements, rather than targeting only the content of CC in the curriculum.

The following steps were undertaken to develop the questionnaire through a consensus process. First, a working group was established to discuss the contents of the questionnaire. The working group was established by the project’s Danish partner, University of Copenhagen, and consisted of five researchers with expertise in different areas in the fields of migration and health. Five relevant domains for investigating CC in medical educational programmes were identified in group discussions: 1) courses and curriculum, 2) teaching staff composition and competencies, 3) resources, 4) management, policies and support and 5) student composition and competencies. Second, input to some of the questions under domain 1 (courses and curriculum) came from a Delphi study that was conducted among researchers of the 12 European partners to develop consensus about key competencies that medical teachers need when teaching CC [24]. Seven key competencies were identified that became the basis of the questions on learning objectives. Additionally, questions for the other domains were constructed based on the literature and other similar surveys and frameworks [20, 22, 23]. The questions were subsequently discussed in the working group, finalised and sent to the partners for comments. Finally, the questionnaire was approved by C2MEs steering committee.

The questionnaire was developed specifically for this study and it is, to our knowledge, the first study that investigates the level of CC in European medical education programmes across countries and targeting different institutional levels. It has therefore not been possible to validate our questionnaire against similar existing surveys. Due to the small sample size further validation was not seen as an option.

The final questionnaire consisted of 32 questions and had three alternative response categories: yes, partly, and no. In order to add a qualitative component to the questionnaire, a free text field was included to allow comments by respondents. All questions had an evidence box to support the informants' understanding of the questions. Eight of the questions pertained to learning outcomes related to CC in the medical curriculum (Table 1), five questions pertained to allocation of resources for CC development and maintenance (Table 2) and six questions pertained to organisational structures, support and policies related to CC within the medical programmes (Table 3). The questionnaire was sent by email to the 12 European partners with a request to forward it to the head of the medical programme, who was asked to complete the questionnaire themselves or with the help of a relevant representative. Since the questionnaire deals with organisational policies and not personal data, prior ethical approval was not required per the Danish Data Protection Law. However, the parent C2ME study including our survey was approved by the Dutch NVMO Ethical Review Board since the project was coordinated in the Netherlands.

**Table 1:** Learning outcomes related to CCs in the medical curriculum

Questions	Yes (n)	Partly(n)	No (n)	Missing	Total
The curriculum includes learning outcomes on knowledge about key social science concepts including “culture” and “ethnicity”	7	3	2	0	12
The curriculum includes learning outcomes on knowledge of how social and cultural factors can affect health, health related behaviors, and healthcare	10	1	1	0	12
The curriculum includes learning outcomes on knowledge about key patient population groups to be identified for any local site	1	6	5	0	12
The curriculum includes learning outcomes on awareness of implicit attitudes, including how one’s own norms, values and biases may affect health care provision	4	5	3	0	12
The curriculum includes learning outcomes on awareness of how culture shapes individual behaviour and thinking (including the cultures of medicine	5	4	3	0	12
The curriculum includes learning outcomes on abilities to work effectively with an interpreter	2	7	3	0	12
The curriculum includes learning outcomes on abilities to identify and take into account socio-cultural factors that may influence patient care	6	5	1	0	12
The programme includes evaluating the students in the 7 key CCs mentioned above	3	8	1	0	12

**Table 2:** Allocation of resources for CC development and maintenance

Questions	Yes (n)	Partly(n)	No (n)	Missing	Total
The organisation and/or the medical education programme has earmarked finding for activities related explicitly to support cultural diversity among students	5	3	5	+ 1	13
The medical education programme has specific resources for development of courses and production of CC related to teaching material and tools	2	5	5	0	12
The medical education programme provides specific resources for international exchange of staff members	3	4	5	0	12
The medical education programme provides specific resources for development and maintenance for websites related to CC	2	1	9	0	12
The medical education programme provides specific resources for experts consulting regarding CC	2	2	8	0	12

**Table 3:** Organisational structures, support and policies related to CC within the medical programs

Questions	Yes (n)	Partly (n)	No (n)	Missing	Total
The medical education programme strategy shows commitment to CC values	2	6	4	0	12
The host organisation (institutional framework such as a hospital, university etc.) of the educational programme strategy shows commitment to CC values	4	6	2	0	12
The strategy is supported by key stakeholders within and outside the medical education programme	4	2	5	1	11
The organisation has appointed a special curriculum advisor to support CC in the curriculum	2	1	9	0	12
The organisation strives to create an environment which is inclusive for all employees and students regardless of cultural background	5	5	1	1	11
The organisation has guidelines, a dispute office or another type of protocol for handling complaints from students experiencing discrimination	8	1	2	1	11

### Survey response and analysis

We received a total of 12 completed questionnaires from the 12 European partners, which described 12 different medical education programmes in ten countries in North, South, East and Western Europe (the Netherlands, Denmark, the UK, Hungary, Germany, Ireland, Spain, Switzerland, Belgium and Norway). These 12 questionnaires form the data basis of our analysis.

The results were analysed through simple descriptive calculations supplemented by quotations of special relevance from the free text fields. Quotes were chosen that either elaborated or nuanced the quantitative responses. The analysis of the free text field was based on a framework method, an approach which involves a systematic process of sifting, charting, and sorting material according to key issues and themes [25]. The framework was based on the questionnaire domains and thereby included the following key themes: course & curriculum, teaching staff composition & competencies, resources, management, policies & support and students composition & competencies. The free text field was coded using these categories and quotes were noted in a simple matrix allowing for intercase analysis per medical school as well as intracase analyses per key theme.

## Results

### Course and curriculum

Over half of the programmes participating in our study(7/12) reported that their curriculum encompasses learning outcomes on key social science concepts, including ‘culture’ and ‘ethnicity’, and 10 of the participating programmes include elements on how social and cultural factors can affect health, health-related behaviours and healthcare (Table 1). However, only one-third (4/12) of the participating programmes have a curriculum that address how medical professionals’ own norms and implicit attitudes may affect healthcare provision. Only two of the 12 participating programmes have a curriculum element that includes learning outcomes on the ability to work effectively with an interpreter. When asked if they evaluate the students on the seven key cultural competencies mentioned in the questionnaire, most respondents (8 out of 12) answered ‘partly’ and one answered ‘no’.

### Teaching staff composition and competencies

Almost all of the participants (11/12) do not monitor or evaluate CC among the teaching staff (not shown). One participant reported: ‘*This is not explicitly monitored or evaluated. Students are invited to comment on their experience of being taught and tutored and may contribute with their own comments on this, although whether they do this and the nature of their comments is not separately extracted’*. Additionally, only one of the programmes offers CC training for the teaching staff. One participant commented that there were only a *‘few initiatives and not from a structural basis but from personal initiative*’. Another participant elaborated: *‘While we offer to teachers the possibillity to aquire a basic qualification in teaching (by training or by assembling a portfolio), CC training or attention to CC is not a part of this’*.

### Resources

The majority of the participating programmes (10/12) allocate few if any resources to the development of courses and teaching materials in CC (Table 2). Looking at resources for international exchange programmes for teaching staff, more than half (7/12) of the participating programmes reported that they have complete or partial funding for this activity. One participant commented that *‘the administrative resources exist but it needs more economic support’* and that *‘unfortunately, there is little demand among staff’*. Another participant reported, *‘There are resources for support for the international exchange of staff members, but these are generally unknown to teachers’.* Furthermore, ten of the 12 participating programmes have no resources allocated for the development or maintenance of webpages about CC, and only two have resources for expert consulting on CC. Three programmes reported in the free text field that they use in-house experts who are involved in advising on CC activities.

### Management, policies and support

Only two of the 12 participating programmes have a strategy that demonstrates a commitment to CC values (Table 3). One participant commented: *‘This commitment is written on paper, so in theory there is a commitment, but it is not translated into real practice’.* Two programmes have appointed a special curriculum supervisor to include CCs. Further, ten programmes reported that they fully or partly strive to create an environment that is inclusive of all employees and students regardless of cultural background. However, only eight programmes in our study indicated that they have guidelines, a protocol or a dispute office to handle complaints of discrimination.

### Students composition and competencies

Only one of the participating programmes stated that their students acquired the ability to be aware of their own cultural bias through their programme (not shown). The majority (7/12) of the programmes declared that they did *not* provide students with adequate CC for future jobs. Only one of the participants said that their programme stengthens the students’ CCs. Comments from the informants explained that *‘Only few students develop these competencies, but more on an individual basis, not because of the education program’.* Another reported that ‘*without formal evaluation related to CC, we cannot adequately answer this question’*… and *‘Some subjects and individual initiatives try to do it, but CC is not considered a transversal competence of every subject, neither is there a structural program which supports it’*.

## Discussion

Our findings provide a snapshot of CC in 12 EU medical educational programmes which represent 4% of all EU medical educational programmes. This snapshot suggests that CC is not adequately included in participating EU medical educational programmes, students are not evaluated on CC, most participating medical programmes do not offer CC training for teachers and resources spent on initiatives related to CC are few. Although participating medical educational programmes were diverse (e.g., drawn from 10 countries throughout Europe) these findings may not be fully representative of all EU medical educational programmes. Future studies are therefore needed to confirm these findings. Furthermore, most of the programmes in our study acknowledge that CC training is not adequate for future healthcare jobs in their respective country (e.g., most medical programmes do not train students to work effectively with an interpreter). Our findings are consistent with previous literature that argue that European medical programmes must become more culturally competent in order to prepare medical doctors to deliver the best possible healthcare in an increasingly diverse population [15, 19, 26]. This is more relevant than ever in light of ongoing and expected increasing global mobility [27].

Although our results show that our respondents’ programmes have incorporated competence on how to handle social determinants of health in their programmes to some extent, this seems not to be the case with CC. This gap indicates how migrant-based determinants and ethnicity are often ignored among the social determinants of health that strongly interact with others [28]. The data also show several gaps and shortcomings in the participating programmes regarding deficient strategies on how to include training in CC for students and teaching staff.

Very few of the programmes in our survey had defined learning outcomes directly related to working with an interpreter. Studies have shown that interpretation is important: it strengthens the communication between doctor and patient and increases equality in access to healthcare and thereby equity in health [29, 30]. The EU-financed project EUGATE, ***‘****Best Practice in Health Services for Immigrants in Europe****’*** from 2009 conducted a Delphi study of expert opinion on what constitutes the best healthcare for migrants and ethnic minorities in Europe**.** The study showed that one of the most important issues in providing healthcare to migrants and ethnic minorities is interpretation [31]. However, lack of sufficient competence regarding interpretation can be seen as an extreme indicator of insufficient sensitivity of medical staff, not least among those working at community health level. This is especially relevant because the new migration flows are making imperative the work of medical professionals and other health professions outside mainstream healthcare organisations.

### Methodological limitations

This is the first study that has investigated how CC is addressed in European medical schools on different institutional levels. However, there are several limitations. First, our study only investigates 12 medical programmes representing 4% of European medical schools and the findings therefore only give a snapshot of the whole picture. Second, the partners participating in the C2ME project were chosen because of their competence and expertise in migrant health research and CC training. We may therefore assume that medical school programmes have somehow benefitted from these experts and that the programmes in the study might show better results than the general pattern among medical programmes in Europe when it comes to CC training. Thereby, the general situation in the medical education in Europe could be even less favourable than our results document. Third, the respondents may have wished to demonstrate a more positive picture of their programme on these often sensitive issues. (In other words, they may have provided ‘politically correct’ responses.) This would add to the risk that the real situation is even more serious. Fourth, the respondents were heads of the medical programmes (and possibly their immediate staff), which may obscure the fact that knowledge about CC throughout their organisations may vary. Fifth, our survey was not previous validated and we could not validate it in our study due to the small sample size.

### Implications

A study by Seeleman et al. [32] argues that the entire healthcare system needs to become culturally competent. It is not sufficient to educate only culturally competent medical doctors because patients also meet other professional groups like nurses, physical therapists and secretaries in the healthcare sector, and they use cafeterias and other services at the hospitals. The healthcare organisation needs to be inclusive with regard to an increasingly ethnically mixed patient population, and the culturally competent practice of individual professionals needs to be supported by managers, organisational strategies and general health policies.

Therefore, we need to broaden the implementation of CCs to include the whole organisation or institution, which means that CC in curricula should contain equity organisational standards. In this regard, Suarez Balcazar et al. [33] state that medical staff and the other professionals in healthcare organisations should be capable of promoting (a) the capacity to adopt a multicultural mission that embraces equality and diversity as values; (b) services and organisational processes that are adapted to the needs of multicultural collective groups; (c) horizontal and reciprocal relationships by including users in the decision-making processes; (d) the capacity to engage in new roles, (e) pluralistic leadership capable of equally representing the needs and views of all constituents and (f) quality and systems change rather than pursuing a quick fix approach, i.e., seeking to maintain services and practices that support multicultural populations.

Finally we recommend that our questionnaire should be validated in order to obtain more robust findings and also to encourage other researchers to use it in assessments of CC in medical education.

## Conclusions

We set out to explore existing CC at different organisational levels in the 12 European University medical education programmes that participated in our study. Our results indicate possible deficiencies in the commitment to and practice of CC within these educational programmes. In light of the accumulating research on the need for cultural and broader diversity competence in healthcare – both structurally and professionally – there is a clear potential for improvements regarding CCs in these medical programmes. Key challenges are to make lasting changes in the medical education programmes by incorporating cultural diversity. These policy changes could lead to a curriculum that promotes students’ awareness of their own biases without promoting cultural stereotypes, and could also create motivated and engaged stakeholders (teachers, management, etc.) within the organisation who are ready and able to promote and allocate resources to CC training for teachers. Medical deans and directors are key actors in the process of changing the focus of educational policies, definition of targets and outcomes, training of teachers and assessing the effect of such changes on students.

## Abbreviations

C2ME: Referring to the project Culturally Competent in Medical Education
CC: Referring to the concept Cultural Competence
EU: Referring to EU and the EEA countries and Switzerland (EU28)
TACCT: Referring to Tool for Assessing Cultural Competence Training
